# Facing the challenge of sustainable bioenergy production: Could halophytes be part of the solution?

**DOI:** 10.1186/s13036-017-0069-0

**Published:** 2017-09-01

**Authors:** Ahmed Debez, Ikram Belghith, Jan Friesen, Carsten Montzka, Skander Elleuche

**Affiliations:** 1 Laboratoire des Plantes Extrêmophiles (LPE), Centre de Biotechnologie de Borj-Cedria (CBBC), BP 901, 2050 Hammam-Lif, Tunisia; 2 Arab German Young Academy of Sciences and Humanities (AGYA), Working group “Energy, Water and Environment”, at the Berlin-Brandenburg Academy of Sciences and Humanities, Berlin, Germany; 3 Department of Catchment Hydrology, Helmholtz Centre for Environmental Research - UFZ, Permoserstrasse 15, 04318 Leipzig, Germany; 4 Institute of Bio- and Geosciences: Agrosphere (IBG-3), Forschungszentrum Jülich GmbH, Leo-Brandt-Str, 52425 Jülich, Germany; 5 Institute of Technical Microbiology, Hamburg University of Technology (TUHH), Kasernenstr. 12, 21073 Hamburg, Germany; 6 Present address: Miltenyi Biotec GmbH, Friedrich-Ebert-Straße 68, 51429 Bergisch Gladbach, Germany

**Keywords:** Biofuels, Biomass, Enzymes, Lignocellulose, Saline environments, Saline and sodic soils

## Abstract

Due to steadily growing population and economic transitions in the more populous countries, renewable sources of energy are needed more than ever. Plant biomass as a raw source of bioenergy and biofuel products may meet the demand for sustainable energy; however, such plants typically compete with food crops, which should not be wasted for producing energy and chemicals. Second-generation or advanced biofuels that are based on renewable and non-edible biomass resources are processed to produce cellulosic ethanol, which could be further used for producing energy, but also bio-based chemicals including higher alcohols, organic acids, and bulk chemicals. Halophytes do not compete with conventional crops for arable areas and freshwater resources, since they grow naturally in saline ecosystems, mostly in semi-arid and arid areas. Using halophytes for biofuel production may provide a mid-term economically feasible and environmentally sustainable solution to producing bioenergy, contributing, at the same time, to making saline areas – which have been considered unproductive for a long time – more valuable. This review emphasises on halophyte definition, global distribution, and environmental requirements. It also examines their enzymatic valorization, focusing on salt-tolerant enzymes from halophilic microbial species that may be deployed with greater advantage compared to their conventional mesophilic counterparts for faster degradation of halophyte biomass.

## Background

Meeting the significant increase in global demand for energy commensurate with the rising population and economic activity represents a major challenge. At current levels of consumption, the world’s energy demand will range between 15 and 18 billion tonnes of oil equivalent (TOE) in 2035, representing a 50% increase as compared to 2009 [1]. These estimations of the International Energy Agency (IEA) imply a marked imbalance between the rate of population increase (1.3-fold) and that of energy consumption (2-fold) between 2009 and 2050 [2].

The limited availability of fossil fuels for producing energy, chemicals, and other materials demands technological development to exploit alternative and renewable sources of energy. As alternative and green sources, wind, solar, and geothermal energy have been commanding increasing attention but these resources cannot meet the demand for bulk chemicals and material resources. An additional source of renewable energy is plant biomass [3]. The carbohydrates in plant biomass are versatile, being important not only for the production of bioethanol but, as oligosaccharides, also as therapeutic agents, e.g. cyclodextrins in the pharmaceutical industry and biomedicine [4]. Agricultural residues and plant waste streams that do not serve as feed or food are of commercial and scientific interest as a source of cellulosic ethanol [5]. Conventional crops, besides being part of the above-mentioned competition for land between food and fuel, always use fresh water, which enlarges substantially the water footprint of bioenergy products from plants [6].

The production of biofuels is mainly based on conventional crops such as sugar cane and maize (*Zea mays*) or on straw. However, sustainable production of bioethanol and biogas from renewable plant resources would globally reduce the dependence of some countries on imported fossil fuels and would have a positive impact on climate change [7]. Starchy biomass (sugar beet and maize, for example) were the first-generation sources of biofuels whereas non-edible lignocellulosic plant material (straw and wood chips, for example) are the predominantly used second-generation sources of biofuels.

Saline areas, generally considered as marginal zones with extremely low productivity, are rich in halophytic vegetation (plants that prefer salt-rich environments), which is able to sustain itself despite high salinity levels. Unlike glycophytes (plants that are affected even by low levels of salinity), halophytes can withstand and even require salinity for optimal growth: this ability or requirement is the result of a complex strategy that integrates different mechanisms at cellular, tissue, and whole-plant levels. Halophytes are useful in many ways: as sources of fibre, oil, and medicines, for landscaping (as ornamental plants), in phytoremediation and medicinal applications, and as oilseed species [8–12]. Although currently halophytes are exploited only on a small scale, their natural habitats occupy relatively large areas throughout the world. Halophytes are becoming increasingly important not only because of the need to avoid the competition for land between food and other uses –– given that fresh water and arable land are in limited supply –– but also because agriculture is being threatened by the steady increase in the extent of saline soils [13]: saline soils cover about 8% of the global land surface, including deserts and salt lakes that are unsuitable for large-scale biomass production.

Areas most suitable for the cultivation of halophytes are semi-arid coastal regions as well as regions where saline water is plentiful either naturally (because of high groundwater levels when the groundwater is saline or brackish) or as a by-product. Introducing halophyte species as new crops that are irrigated with saline water is becoming an increasingly attractive, feasible, and sustainable option for ensuring food security in several salt-affected regions [14, 15]. Consequently, economic utilization of halophytes is receiving increasing attention, especially in arid regions where intensive irrigation or shortage of fresh water forces people to use marginal resources such as brackish underground water [16]. Introducing halophytic plants could be advantageous because they are cheaper to grow and naturally abundant on saline soils and thus do not have any adverse impacts on the human food chain [17]. High-yielding non-food biomass crops are being currently promoted worldwide to meet the increasing demand for energy and to contribute to reducing the emissions of greenhouse gases. Another major advantage of halophytes is their ability to produce satisfactory yields even under adverse conditions as well as to serve as a sustainable and direct source of income for farmers [18]. Most salt-tolerant halophytes accumulate high levels of salt in their shoots. Although such high levels of salt do not impair biomass production [5], they have other undesirable consequences such as slowing down the degradation of biomass feedstock for bioenergy production by inhibiting the enzymatic decomposition of lignocellulose and accelerating the corrosion of reactor components. Measures to overcome these effects include adjusting the organic loading rate in the reactor or co-digestion with conventional plant materials that serve as energy sources [19]. Another option to increase bioethanol yield is to apply salt-tolerant enzymes from halophilic microorganisms to degrade lignocellulose under saline conditions.

**Fig. 1:**
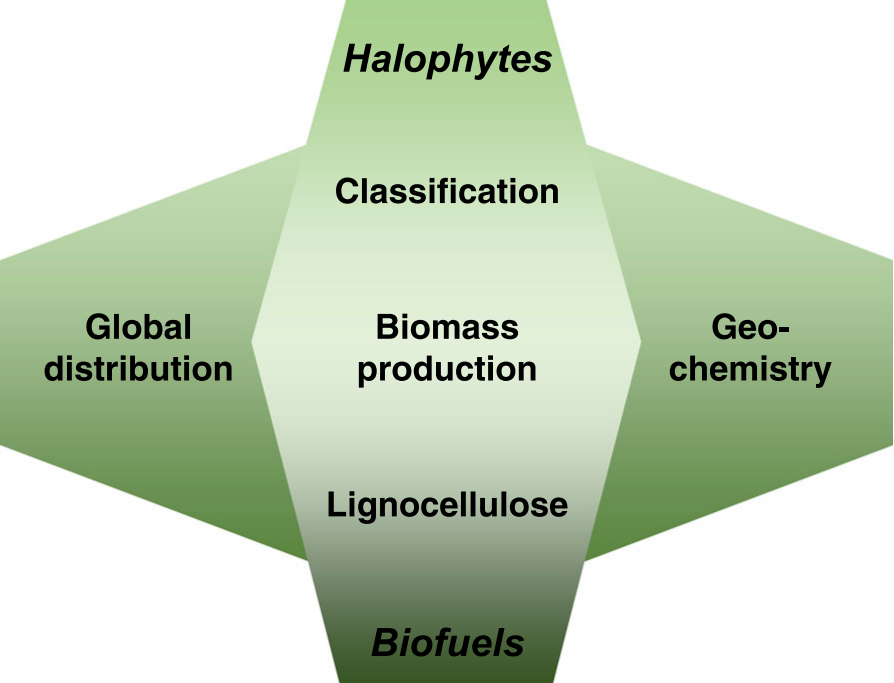
Structure of the review. Chapters deal with the classification and distribution of halophytes including data on soil chemistry. Examples of halophytes with a potential for bioenergy production are given and the process of biomass degradation is described in detail

This interdisciplinary review provides an overview of halophytes as potential sources of energy, their availability on the scales necessary for producing bioenergy, and the technical challenges related to processing the lignocellulose from salt-containing biomass (Fig. 1). To deeper address these topics, Section 1 provides a general classification of halophytes and a description of their natural habitats. Section 2 describes halophyte habitats starting with the types of habitats, assessment and monitoring methods for saline soils, and the global distribution of saline environments and also emphasizes the processes of soil salinization and soil chemistry of saline environments. Section 3 identifies halophytes species that are exclusively suitable for biofuels production, whereas Section 4 summarizes the process of enzyme-mediated degradation of cell walls in plants, focusing on salt-tolerant enzymes to improve the efficiency of converting biomass into bioenergy. Section 5 concludes the review and provides perspectives for future research on treated and produced water for halophyte irrigation, on side effects of cultivating halophytes, and on improving bioenergy production from halophytes.

## Classification of halophytes

Halophytes represent a phylogenetically heterogeneous group of extremophilic plants native to saline habitats, which can cope with or even require high levels of salt for optimal growth [20]. Halophytes are present in nearly half of the plant families. Currently, more than 2600 halophyte species, distributed worldwide, have been identified, which reflects their potential as cash crops under saline conditions [21, 22].

**Fig. 2:**
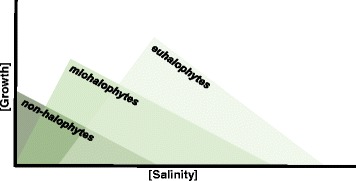
Preferred salt level of different plant types. Euhalophytes prefer higher salt concentrations than miohalophytes, whereas non-halophytes can tolerate only low levels of salts. Details are given in the text and in Fig. 4

Halophytes were defined as salt-tolerant plants that can thrive and complete their life cycle in habitats with soil salinities up to 200 mM NaCl [23]. More recently, halophytes were described as plants that can tolerate salt concentrations ranging from 500 mM NaCl to 1000 mM NaCl [21]. Halophytes are generally classified into two categories: euhalophytes, considered the most tolerant species, which *require* salt for optimal growth and have the ability to grow at concentrations up to 500 mM NaCl, and miohalophytes, considered less tolerant, that do not show salt-induced growth stimulation [24, 25] (Fig. 2). The ability of halophytes to survive under such challenging conditions is complex and multifaceted, but it closely depends on the plant ability to control salt uptake through the compartmentalization of Na^+^ (‘includer’ species) and/or to salt extrusion (‘excluder’ species).

## Habitats of halophytes

The description of different halophyte habitats, and of their distribution and functioning, is essential for assessing the potential of halophytes as sources of bioenergy. This section, therefore, focuses on the soil–plant relationship in salty environments. The physical chemistry of soils and sediments needs to be discussed and the soil salinization process is described to more accurately assess the untapped potential of this ecological niche for producing biomass using halophytes.

### Types of saline environments

Typical halophyte habitats are tidal flats in coastal regions, as they are prone to saltwater intrusion, and saline and sodic soils. Besides these ecosystems or habitats, sources of saline water, such as treated effluent from wastewater treatment plants and water from oilfields, also provide habitats for halophytes.

**Fig. 3:**
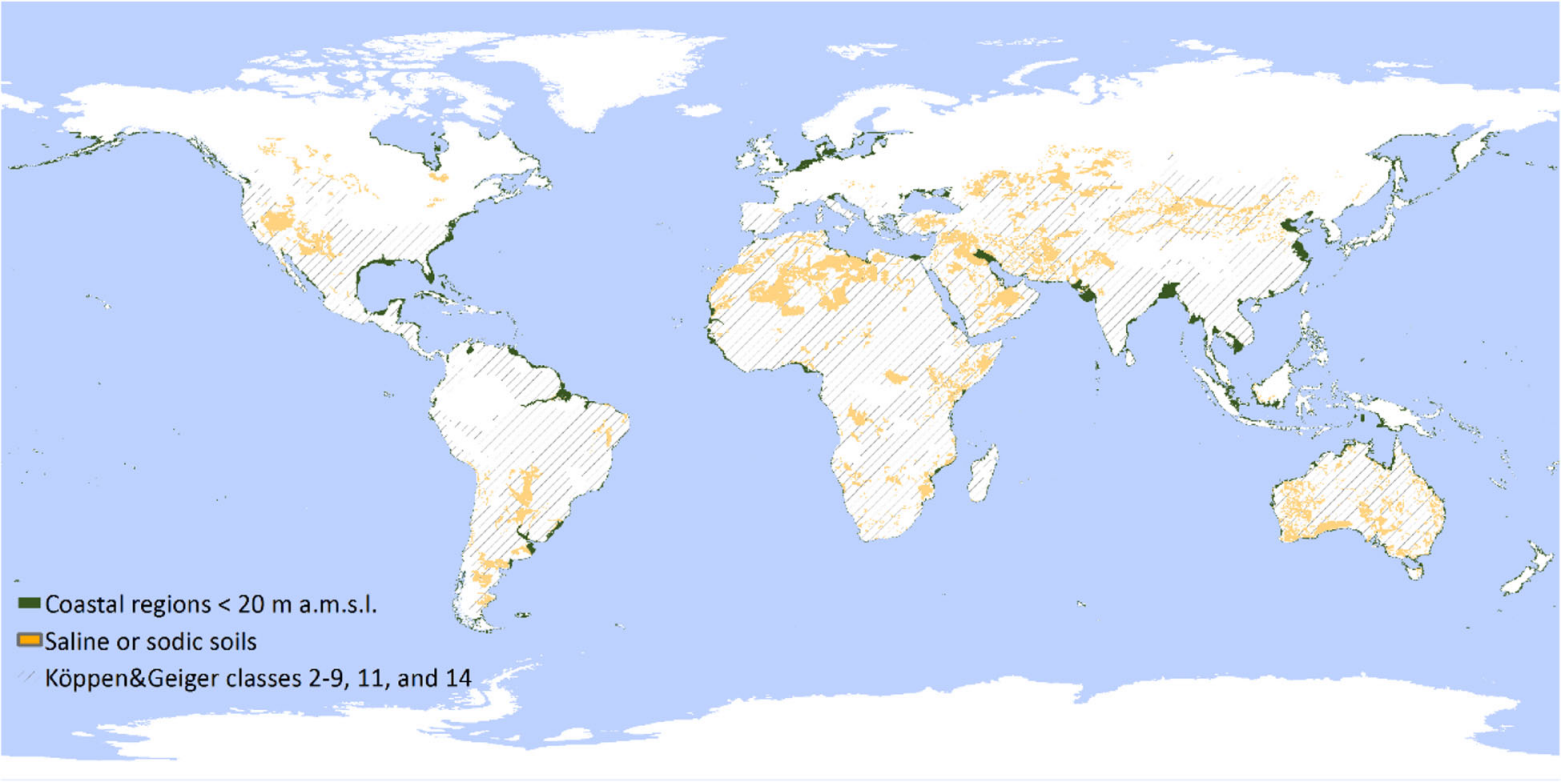
Distribution of saline environments. Coastal regions 20 m below the mean sea level were mapped using Shuttle Radar Topography Mission (SRTM) and Moderate-Resolution Imaging Spectroradiometer (MODIS) land/sea mask data using Google Earth and ArcGIS [129, 130]. The distribution of saline or sodic soils was taken from the Harmonized World Global Soil Database [30]. The Koeppen and Geiger (classes Am, Aw, B, Csa, Csb, Cwa, and Cfa) data show tropical to (semi-)arid regions with dry climate or high seasonality [131]

Tidal flats are regions that are flooded during the high tide and exposed during the low tide [26]. Although global data on tidal range as well as on elevation are available, the mapping of coastal regions is somewhat limited because it requires highly accurate data. Regions prone to saltwater intrusion are coastal regions that exhibit a high groundwater abstraction as well as estuaries or regions with irrigation or drainage channels that act as conduits for saltwater. Saltwater intrusion is defined as the mixing of saline water with freshwater aquifers. Global mapping of saltwater intrusion continues to be a challenge because the intrusion depends on the rates of groundwater abstraction and natural groundwater recharge as well as on the geology and hydrochemistry of coastal groundwater. To show the extent of coastal regions prone to saltwater intrusion, Fig. 3 includes regions 20 m above the mean sea level. Although not linked to saltwater intrusion, the limit was set at 20 m because it matches the approximate maximum depth of hand-dug wells (www.sswm.info/content/dug-wells), which enables such wells to be used as sources of irrigation water that can be pumped at relatively low costs in most regions of the world, thereby enhancing saltwater intrusion. Coastal regions are being increasingly salinized because of anthropogenic activity (increased demand for water) and because of sea level rise due to climate change, reduction of river flows, and greater frequency of extreme events (e.g. storm surges) [27, 28].

Salt-affected soils are those with high contents of soluble salts, which restrict normal plant growth. Besides typically saline or sodic soils, such as Solonchakz or Solonetz, soils are considered saline or sodic if their electrical conductivity is higher than 4 dS/m or 6% saturation with exchangeable sodium [29]. The distribution of salt-affected soils can be determined using global soil maps, such as the Harmonized World Soil Database [15, 30].

In water-scarce regions, re-use of water resources is emphasized; as a result, urban wastewater is often used for irrigation. The so-called ‘treated effluents’ (TE) are available from the household level to village level and city level and are often highly saline [31]. Generally, TE are mixed with fresh water to achieve usable salinity levels, but such mixing would not be required for halophytes. The overall quality of TE depends to a great extent on the quality of municipal water supply, the nature of wastes added during use, and the extent to which the wastewater is treated [32]. However, salt concentration in TE is often higher than in the original source, posing a greater risk of aggravating soil salinization. Assouline and Narkis [33], for example, showed that long-term effects of irrigation with TE were a significant degradation of soil structure and hydraulic properties due to the increased percentage of exchangeable sodium.

**Fig. 4:**
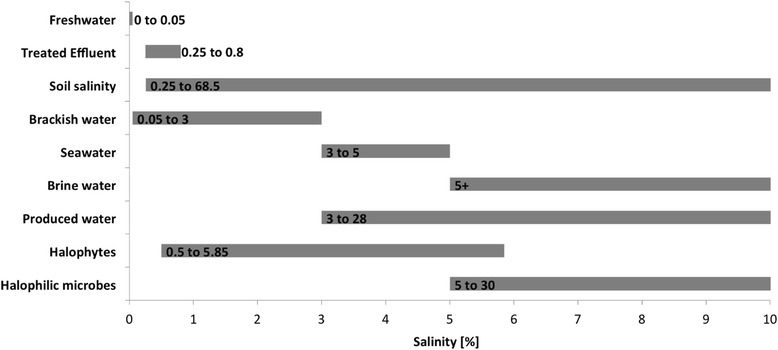
Salinity classes for different water types

As with TE, water from oilfields is not linked to specific regions or ecosystems. Both are mentioned because they are sources of highly saline water (Fig. 4) available in abundance. Water thus produced is pumped out during oil production and can be as much as ten times the volume of oil produced. Although up to 60% of such water is re-injected into the wells to maintain aquifer pressure, huge amounts remain on the surface and have to be suitably disposed of, which is where halophytes are proving useful [34, 35].

### Monitoring and assessment of salt-affected soils

Soil salinity is typically defined as the concentration of dissolved mineral salts in soil or, more specifically, in soil solution [36]. The major cations are Na^+^, Ca^2+^, Mg^2+^, and K^+^, and the major anions are Cl^−^, SO_4_^2−^, HCO_3_^−^, CO_3_^2−^, and NO_3_^−^. Further contributions to soil salinity come from B, Sr^2+^, SiO_2_, Mo, Ba^2+^, and Al^3+^. Soil salinity is expressed in millimoles per litre or in micromoles per litre, but more often as a combined measurement using electrical conductivity (EC) of the solution reported in millisiemens or decisiemens per metre. To measure salt content in a laboratory in addition to EC, total dissolved salts (TDS) are measured by evaporating a known volume of water and weighing the remaining solid residue. Electrical conductivity can be measured far more quickly because it is based on the difference in EC between saline and non-saline water: a current is applied to two electrodes immersed in the water sample; the current flow between the two electrodes changes depending on the salt content. Typical units (including conversions) are 1 dS/m = 0.064% = 0.64 g/L = 640 ppm = 11 mM NaCl.

However, soil salinity is dynamic and varies with soil water content. Therefore, EC measurements are typically performed in soil samples saturated to reference water content (ECe). Soils with ECe higher than 4 dS/m are classified as saline, and those with ECe less than 4 dS/m are classified as non-saline. Soils with ECe less than 4 dS/m could also be sodic, when the content of exchangeable Na^+^ is 15% more than the cation exchange capacity (CEC). Soil maps are usually based on soil profiles. Samples from such soil profiles are analysed in the laboratory and a number of physico-chemical properties are determined, including the content of solutes such as salt [37]. Next to laboratory analyses, in situ observations, such as EC, are usually taken. These measurements are classically done on a point scale (soil) or from water samples (such as well observations). Salinity can also be measured remotely, using geophysical techniques such as electromagnetic induction (EMI) or electrical resistivity tomography (ERT), either on the ground or from the air, using a helicopter or fixed-wing aircraft [37–40]. Moving to larger scales, satellite data are used as well through relationships between hyperspectral or radar wavebands and salinity or dielectric properties [39, 41, 42].

### Mapping of saline environments

The global extent and distribution of saline environments can be assessed using global soil data sets such as the Harmonized World Global Soil Database [30]. The global distribution shows that many regions are at continental locations and often in very hot and arid regions, unsuitable for agriculture (Fig. 3). In addition, many of the continental saline environments are very sparsely populated (Fig. 3). With respect to coastal regions, saltwater intrusion continues to affect increasingly larger areas, especially regions with high population densities; 44% of the global population lives within 150 km from a coast (*United Nations Atlas of the Oceans*, www.oceansatlas.org) [43]. In addition to the densely populated areas that are affected because of anthropogenic saltwater intrusion following overexploitation of groundwater aquifers, sea level rise also makes low-lying coastal regions more prone to salinization [27, 28]. Areas prone to saltwater intrusion (Fig. 3.) account to about 240 Mha, which corresponds to 1.8% of the global land surface area. Mapping saline environments on a large scale is usually done through soil maps, e.g. national or global soil maps. On regional and smaller scales, remote sensing has been used for mapping salinity [44]. However, global mapping approaches are limited because of different types of halophyte habitats and inadequate data quality on a global scale [15, 45]. Global studies estimate the extent of salt-affected land at 1128 Mha; in other words, about 8.5% of the global land surface area is saline or sodic [15, 46].

With regard to halophytes for bioenergy, many studies estimate the extent of halophyte habitats in combination with plant growth models and economic analyses [14, 15, 19, 47]. In China, for instance, bioenergy crops are cultivated extensively mainly on coastal saline lands so that arable farmland can be used for grain crops [47]. In addition, on a 2.0 million ha large tidal flat, the area under halophytes is increasing at rates ranging from as low as 1.3–2.0 ha per year to as much as 10,000 ha per year to meet the increasing demand for bioenergy due to large-scale industrialization. These non-agricultural coastal lands are suitable for halophyte development but not for the traditional grain crops because the lands are extremely saline and because of other associated constraints such as drought and nutrient deficiency [48].

### The process of soil salinization

Soil salinization – high concentration of salts on or near the soil surface – is a chronic problem in many arid and semi-arid regions where evapotranspiration (ET) exceeds rainfall [49]: ET removes pure water as vapour from soil, concentrating the salts in soil solution. However, humid climates are not free of salinization either.

‘Primary salinization’ occurs in arid and semi-arid climatic zones. In addition to geochemical weathering of minerals present in rocks, a distinction is drawn between salt supply by precipitation and that from groundwater: the former contains dissolved salts from the atmosphere mainly originating from the sea, the so-called atmogene salts. Quickly leached in humid climates, these atmogene salts typically accumulate in soil in arid or semi-arid climates. Therefore, desert soils are typically salty. The amount of accumulated salts depends on the distance from the sea, rate of precipitation, duration of arid conditions, topography, and conductivity of soil water. Groundwater salinization in humid climates occurs only in those areas that are close to the sea, e.g. in marshlands without any dykes or ditches for drainage. In arid climates, groundwater-influenced soils are often enriched with salts. The rise of groundwater through capillaries brings the salts upwards from the deeper layers; once the water evaporates, salts accumulate in the surface layer according to their chemistry: CaCO_3_ at lower levels; gypsum, sodium carbonate and Na_2_SO_4_, at intermediate levels; and finally Na^+^ and Ca^2+^ and nitrates at the upper levels. This can lead to salcretes in the subsoil or salt crusts at the soil surface.

Artificial soil salinization as a consequence of direct human activity is referred to as ‘secondary salinization’. Salinization was earlier thought to be an environmental problem restricted to arid regions, whereas now it is recognized as a global environmental concern affecting humid regions as well—a result of artificial inputs in the form of road de-icers, sewage, and water softeners [50]. However, the most severe impacts of salinity on agriculture occur in arid and semi-arid regions. Climate change, with the predicted hotter and drier conditions, will aggravate the problem of salinity in many regions, together with the impacts of such anthropogenic activities as the use of marginal water sources, saltwater intrusion due to overexploitation of coastal aquifers, and rapid and unsustainable withdrawal of water from inland aquifers before they are fully recharged [49]. Salts enter soil through diverse pathways, but the main driver of anthropogenic salinization is the misuse and mismanagement of rapidly expanding irrigation [51]. Artificial raising of the water table due to inadequate drainage can also make soils saline, similar to the process of primary salinization [52]. Highly saline drainage water carries the risk of contaminating or even degrading the associated groundwater and surface water.

Disturbance to natural vegetation can lead to a hydrologic imbalance between precipitation and ET. Replacing plants that have high rates of ET (forests) with plants that have low rates of ET (crops) in tracts in which the water table is high, e.g. by permitting overgrazing on pasture lands, the groundwater may rise further, bringing with it dissolved salts, which may accumulate close to the soil surface forming saline or sodic soils. Intensive agriculture with expanded irrigation as well as using marginally saline water for irrigation will affect an already fragile environment and could threaten the sustainability and functioning of such agro-ecosystems [51].

In general, soil salinization has been shown to be reversible under an efficient drainage network [53]. In such cases, investing in an efficient drainage system may pay off: halophytes may be cultivated initially, when salinity levels are high, for biofuel production, until the desalination strategy is successful in restoring the soil so that crops can be cultivated for food, supported by judicious irrigation. A multi-year rotation could be established that alternates between halophytes and crops.

### Physical chemistry of soils and sediments in saline environments

Salinization in irrigated agricultural soils is typically a process that takes years or decades. In order to analyse the altered physical chemistry of soils during salinization, several experiments were conducted involving salty or brackish water for irrigation [54]. Among the direct affects was a significant increase in the concentrations of chlorides, sodium, sulphur, and potassium in the free water (water found in soil pores). Salt inputs to the soil solution lead to exchange of cations such as Na^+^, Mg^2+^, and K^+^ for those of Ca^2+^, H^+^, and NH_4_^+^ from the cation adsorption complex of the sediment [54]. In clay minerals, iron can also be mobilized. When salts are present at high concentrations, this exchange occurs rapidly; as a result, calcium concentrations in free water may exceed the initial concentrations [55].

Soil sodicity is often linked to soil salinity. Irrigation with saline water leads to accumulation of monovalent Na^+^-cations in soil solution, which can severely degrade soil structure. Clay particles enter the dispersed phase and the soil may swell, reducing aeration as well as the hydraulic conductivity of the saturated soil significantly and shifting the water retention curve towards smaller pores. The effect of sodicity depends on the type of clay and will be stronger in montmorillionitic soils (consisting of 3-layer clay minerals) than in kaolinitic soils (consisting of 2-layer clay minerals) and more common under tropical climates.

Typical salt-affected soils, according to the World Reference Base for Soil Resources (WRB) soil classification [56], are Arenosol, Calcisol, Gypsisol, Kastanozem, Solonchak, and Solonetz (Table 1). Arenosols (Psamments in the US soil taxonomy) are sandy soils in which soil horizons are poorly differentiated. The geographical distribution of Arenosols is not restricted to the deserts of the world; they are also found in humid climates, e.g. coastal dunes. With almost no natural vegetation, Arenosols are very poor in humus, and arable farming is possible only with intensive application of fertilizers. In desert regions, such soils are often used for irrigated agriculture because the large sand fraction ensures good drainage, thereby lowering the risk of secondary salinization. Solonchaks (Salorthids) are pale or grey salt soils with a thick saline horizon within 30 cm of the soil surface. They develop in low-lying areas with a shallow groundwater table (external Solonchaks): soluble salts are transported through capillary action and accumulate in the topsoil or at the soil surface. Salt crusts may develop to some extent. Alternatively, in soils where the average groundwater level is not that close to the surface, salts can accumulate at greater depths (internal Solonchaks). In the latter case, the source of salt is not typically the groundwater but saline precipitation that occurs only under arid conditions. The sparse natural vegetation leads to low humus content. Artificial Solonchaks develop when salts introduced with irrigation water are not properly removed through drainage. Solonetz soils (Natric great groups of several orders) typically develop from Solonchaks by lowering of the water table, high humidity, or groundwater with high Na^+^ content. The high Na^+^ saturation results in high pH, between 8.5 and 11, and supports the downward movement of clay and humus. The developed clay horizon is characterized by low permeability and low aeration, making such soils particularly unsuitable for cultivation, even for halophytes. Raising crops that are Na^+^-resistant or tolerant could gradually make the soil more permeable and eventually suitable for intensive cultivation. Other salt-influenced soils relevant here are the Kastanozems (aridic borolls and ustolls), steppe soils with massive humus topsoil with lime and gypsum accumulation and soluble salts in the subsoil. Calcisols (Calcids) and Gypsisols (Gypsids) with substantial accumulation of secondary carbonates or gypsum are often salt affected and then form subclasses of Solonchaks and Solonetz, in which solid layers of lime and gypsum in the subsoil often hinder cultivation as well as rooting.

**Table 1:** Salt-affected soils and their suitability for cultivating halophiles for biomass provided adequate irrigation is available [65]

Soil	Geographical distribution	Potential to cultivate halophytes	Comments
Arenosol	Mainly on aeolian, but also on marine, littoral, and lacustrine sands, e.g. in the Kalahari, Sahel, various parts of the Sahara, central and western Australia, the Near East and western China, sandy coastal plains and coastal dune areas	++	High percolation losses during surface irrigation; soil conservation measures necessary
Solonchak	Arid and semi-arid parts of northern Africa, the Near East, former Soviet Union and Central Asia, widespread in Australia and the Americas	+	Irrigation should be accompanied by drainage systems
Solonetz	Semi-arid temperate continental climate, e.g. in the Ukraine, the Russian Federation, Kazakhstan, Hungary, Bulgaria, Romania, China, the United States of America, Canada, South Africa, Argentina, and Australia	+/−	Deep ploughing to improve soil permeability; irrigation with Ca-rich water
Kastanozem	Eurasian short-grass steppe belt, the Great Plains of USA, Canada, and Mexico; pampas and Chaco regions of northern Argentina, Paraguay, and south-eastern Bolivia	+++	Care has to be taken about secondary salinization and wind and water erosion
Calcisol	Often together with Solonchaks in arid and semi-arid tropics and subtropics	+	Amelioration might be necessary to break lime banks
Gypsisol	Kazakhstan, Turkmenistan, Uzbekistan, the Libyan and Namibian deserts, southern and central Australia, and south-western USA	++	Rapid dissolution of soil gypsum may lead to irregular subsidence of the land surface and corrosion of concrete structures

(+++) highly suitable, (++) well suitable, (+) suitable, (+/−) rather unsuitable

In addition to the high impacts of salinity on soil genesis that builds individual salt soils, the nutrient dynamics of free water are altered in all such soils during salinization; therefore, the availability of nutrients to plants also changes. The general pattern depends strongly on soil conditions and is often not explicitly determinable. The studies discussed below attempt to identify a few trends related to the chemistry of soil.

The processes that are affected significantly are denitrification (the reduction of nitrate (NO^3−^) to molecular nitrogen) and nitrification (the oxidation of ammonia (NH_4_^+^) to nitrate), because these are mainly driven by microbial metabolism. In this context, the availability of salt-tolerant microorganisms is particularly important [57]. Increasing salinity and sodicity cause an exponential decline in potentially mineralizable nitrogen [58]. In most plants, total shoot nitrogen uptake decreases under saline conditions because it is accompanied by an increase in chloride uptake [59]. However, sensitivity to salinity depends on the ionic form of nitrogen, whether nitrate or ammonia: nitrate uptake is markedly reduced whereas ammonia uptake is not. In contrast, relative growth rates decrease slightly in nitrate-fed plants but remain unchanged in ammonia-fed plants [60].

Phosphorus concentrations in soil solution are also altered by salinity, but whether they are increased or decreased remains uncertain, because experimental results have been contradictory [61, 62]. However, phosphorus immobilization and sorption are reported to be much stronger than phosphorus mobilization and desorption [54]. In addition to co-precipitation with calcium or calcium carbonate, phosphorus can bind to newly formed iron (hydr)oxides [63]. Therefore, experiments have shown salinity-induced reduction in phosphorus concentrations in plant tissues [64]. On the one hand, such reductions lower shoot growth; on the other hand, they stimulate the formation of root hairs and lateral roots in several plant species [59].

Salinity affects the production of CO_2_ in soil but the estimated emissions differ substantially, so that a clear picture cannot be provided here. However, significantly decreased methanogenesis was documented [54, 65], because in sulphate-rich environments sulphate-reducing bacteria were more performant than methanogenic bacteria when competing for organic substrates [66]. Therefore, biomass for biofuel production on salt-rich soils may have a greater effect on reducing greenhouse gases than that on non-salty soils. Increased N_2_O production under anaerobic conditions and low pH was reported for lab experiments; however, the true ecological relevance of the production of greenhouse gases (CO_2_, CH_4_, and N_2_O) could not be assessed under field conditions [66].

The pool of soil organic carbon (SOC) is dependent on inputs from vegetation, and the effects of salinity and sodicity on plant health adversely impact SOC stocks in salt-affected areas, generally leading to lower contents of SOC [67]. Dissolved organic carbon (DOC) is known to be the most mobile and dynamic among the non-living fractions of soil organic matter and affects many biogeochemical processes such as nutrient translocation and leaching, microbial activity, and mineral weathering [68]. In salt-affected soils, CEC and iron and aluminium concentrations strongly influence DOC sorption. It was reported that DOC loss from saline–sodic soils is lower than that from sodic soils because of cation bridging at high electrolyte concentrations and suggest that increasing the electrolyte concentration in sodic soils by liming or irrigation with saline water may reduce the loss of nutrients through leaching and increase organic matter sequestration [66, 69]. In salt-affected fields where sugar cane cultivated, greater salinity and sodicity decreased soil microbial activity and nitrogen mineralization, but no evidence of accumulation of soil organic matter was found, due to reduced plant growth, which lowered the inputs of organic matter to soil simply because reduced plant growth means less biomass [58].

Another problem related to salt-affected soils is the mobilization of heavy metals. The mobilization of Pb^2+^, Cd^2+^, Cu^2+^, and Zn^2+^ appears to be regulated by several mechanisms, e.g. the competition with Ca^2+^ or Mg^2+^ for sorption sites or the complexation with chlorides or sulphates. Clay and silt soils can reduce such mobilization of Cd^2+^ due to salinization by strongly retaining the fine fractions [70].

A general positive side effect of cultivating halophytes for biomass production is the elimination of herbicides: no other plants can survive in this specific ecological niche.

## Halophytes as potential sources of biodiesel and bioethanol

The lignocellulosic biorefinery concept encompasses the use of biomass to produce biodiesel, bioethanol, biogas, biomethanol, hydrogen, charcoal, and other energy sources [71]. However, this review focuses exclusively on the potential of salt-tolerant plants as a source of biodiesel, and bioethanol. Biodiesel is composed of monoalkyl esters of long-chain fatty acid derived from renewable feedstock as well as vegetable oil and animal fats [72]. Biodiesel is generated from such oil-rich sources as sunflower, soybean, oil palm, rapeseed, and rice bran whereas bioethanol is produced by fermentation and could also be generated from a variety of agricultural wastes.

Extremophile plants such as halophytes are considered promising candidates for large-scale production of bioenergy in the form of bioethanol, and biodiesel. Indeed, biofuel from halophytic biomass may represent a sustainable alternative to conventional fossil fuels in dealing with the global issue of resources for human consumption and the depleting stocks of fossil fuels [17, 47].

### Halophytes as resources for biodiesel

Fatty acid methyl esters from halophytes appear to be an economically attractive source for biodiesel production and could be considered an important substitute in conventional diesel engines [17, 47, 48, 73, 74]. To avoid the competition between bioenergy crops and food crops, a crucial step is to select the cheapest feedstock to produce biodiesel at low cost. In general, feedstock for biodiesel is classified into two categories: edible oils, which are obtained from food crops such as rapeseed, soybean, sunflower, and oil palm; non-edible oils, which are prepared from such non-food resources such as *Jatropha, Pongamia,* sea mango, algae, halophytes, animal fats including chicken fat, and other sources as organic waste or recycled oil [75, 76]. In fact, non-edible oils from non-food resources are assuming a major role worldwide in (i) meeting the increasing demand for bioenergy following large-scale industrialization and (ii) conferring several other benefits, mainly because they use land or water that is unsuitable for crop production, decrease the rate of deforestation, and provide useful by-products. In addition, non-edible oils are economical and more widely available than edible oils [75] (Table 2).

**Table 2:** Examples of biofuel halophytes

	Salt threshold (mM NaCl)	Reference
Halophyte species		
*Aeluropus lagopoides*	150	[133]
*Alhagi maurorum*	500	[84]
*Arthrocnemum macrostachyum*	-	-
*Atriplex nitens*	-	-
*Atriplex rosea*	1000	[84]
*Cakile maritima*	200	[79]
*Climacoptera brachiata*	-	-
*Climacoptera lanata*	470	[134]
*Cressa cretica*	425	[135]
*Cynodon dactylon*	50–150	[136]
*Desmostachya bipinnata*	400	[100]
*Euphorbia tirucalli*	-	-
*Halogeton glomeratus*	100	[137]
*Halopyrum mucronatum*	90	[138]
*Halostachys belangeriana*	-	-
*Haloxylon stocksii*	500	[95]
*Helianthus tuberosus*	50–125	[139]
*Jatropha curcas*	60	[140]
*Juncus maritimus*	150	[141]
*Karelinia caspia*	-	-
*Kosteletzkya pentacarpos*	-	-
*Kosteletzkya virginica*	420	[84]
*Leptochloa fusca*	-	-
*Miscanthus giganteus*	80	[142]
*Panicum turgidum*	0,100, 200	[95]
*Phragmites karka*	500	[95]
*Pongamia pinnata*	150	[143]
*Salicornia bigelovii*	200	[47]
*Salicornia europaea*	500	[144]
*Salicornia fruticosa*	100	[92]
*Sarcocornia ambigua*	500	[91]
*Spartina alterniflora*	100	[145]
*Sporobolus virginicus*	-	-
*Suaeda aralocaspica*	500	[84]
*Suaeda fruticosa*	800	[146]
*Suaeda paradoxa*	-	-
*Tamarix aphylla*	150	[147]
*Typha domingensis*	100	[148]
*Urochondra setulosa*	200	[149]
Conventional species		
*Beta vulgaris*	25	[150]
*Brassica napus*	24	[151, 152]
	40	
*Glycine max*	25	[153]
*Jatropha curcas*	30	[140]
*Panicum virgatum*	0-50	[154]
*Saccharum officinarum*	20	[155]
*Zea mays*	20	[156]

- no information available

It is well known that seeds of numerous halophytes contain significant amounts of oil and may serve as a source of biodiesel [77]. More than 350 oilseed crops are considered potential sources of biodiesel [76]. However, at least 50 species of seed-bearing halophytes are regarded as potential sources of grain and edible oil including those with the highest quality seeds comparable or even better than others seeds that are rich in carbohydrates, protein, and fat [78].

**Fig. 5:**
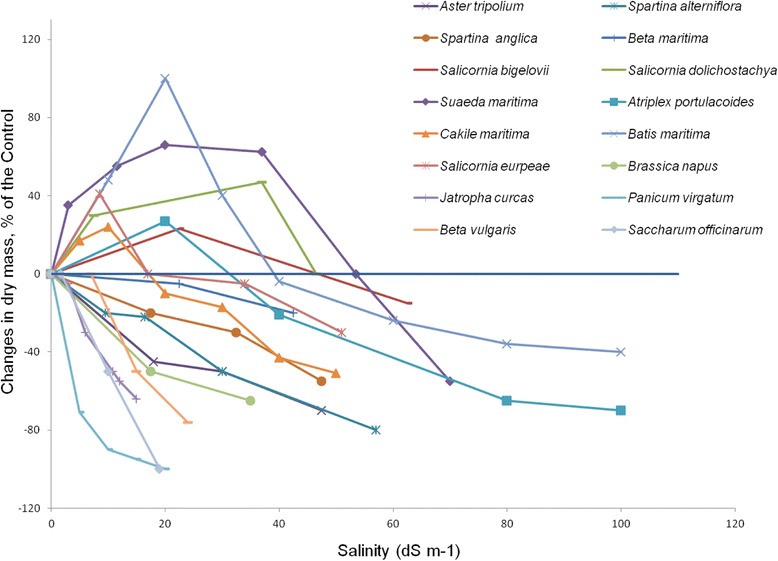
Performance (as per cent of dry mass in the control) of some halophyte species when challenged with increasing salinity (dS m^−1^) as compared to that of conventional crops used for biodiesel production. The figure is a slight modification of that given by [132]

*Cakile maritima* and *Sarcocornia ambigua*, are oilseed halophytes potentially useful in industry [79, 80]. Due to the composition of fatty acid esters extracted from their seeds, these species are promising candidates for biodiesel production [74]. *Sarcocornia ambigua* is a perennial species, belonging to a genus of small, succulent halophytic shrubs, and widely distributed in coastal marshes, mangroves, and salt deserts of South America [81]. *Cakile maritima* is a fleshy-leaved annual species with a major ecological role in stabilizing sand dunes. Seeds of *C. maritima* can germinate in up to 100 mM NaCl and resume germination even after exposure to 200 mM NaCl [82, 83]. Moreover, the plant requires moderate salt concentrations (100 mM NaCl) for maximal biomass production and seed yield, and its oil yield and composition were not affected by up to 200 mM NaCl salinity [64, 79]. These salt-tolerant species usually exhibit optimal growth under salinity levels of 100–200 mM NaCl, unlike conventional biofuel crops such as canola, Jatropha, switchgrass or sugarcena, which appear to be salt sensitive, as reflected by their severely reduced growth even at low levels of salinity (Fig. 5).

A recent study pointed out that the oil content of seeds of halophytes *Salicornia bigelovii*, *Salicornia europaea*, and *Suaeda aralocaspica* is higher than those of *Pongamia pinnata* (18%–27%), soybean (20%), *Jatropha curcas* (29%–38%), and castor (*Ricinus communis*, 30%) [84] Although they are rich in oil, castor, soybean, and *Jatropha* are widely known to be sensitive to salt (Fig. 5). Seed oil content in *Kosteletzkya pentacarpos* is close to 19% [85]. In fact, *K. pentacarpos* yields nearly 1500 kg/ha of seeds with a total oil yield of 330 kg/ha [85–88]. Another facultative halophyte, *R. communis* (*Euphorbiaceae*), with great potential as a good-quality biodiesel feedstock, is found in littoral habitats and salt marshes. Seeds of *R. communis* contain as much as 47%–50% oil and are rich in ricinoleic acid (87%). The latter constitutes the major component of oil, because of which the plant is considered among the most important renewable oil resources [48].

*Salicornia bigelovii*, a halophyte known for its high-quality oil, is a leafless annual with green, jointed, and succulent stems, which grows in salt marshes and is widespread in many countries such as the United States, Mexico, Saudi Arabia, the United Arab Emirates, Egypt, Eritrea, and Pakistan [80, 89]. *Salicornia bigelovii* shoots are thicker and more succulent when the plant is grown at high salinity levels. This phenomenon, which is associated with stimulated growth and water uptake, can be explained by the efficient cellular accumulation of sodium. The fact that *S. bigelovii* seeds contain approximately 28% oil, which is similar to soybean in oil quality and yield [47, 90], makes it one of the most promising oilseed crops. Fatty acids represent about 48% of the total lipids (dry matter content is 21 mg/g) in *Salicornia* spp. shoots, when irrigated with seawater. The composition was found to be constant even when the dry matter was reported to be only 17 mg/g [91]. The latter can also be converted to biodiesel or bio-derived synthetic paraffinic kerosene (Bio-SPK) through trans-esterification [92–94]. *Suaeda fruticosa* is a facultative perennial halophyte, found in inland and coastal salt marshes, salt deserts of Pakistan, and in northern, central, and southern Tunisia. Seeds *of S. fruticosa* have recorded up to 400 mM NaCl during germination. The plant can grow up to 2 m tall. Moreover, *S. fruticosa* can tolerate highly saline substrates (up to 1000 mM NaCl). Its seeds contain approximately 26% oil, which can be used to produce biodiesel [84, 95].

*Cressa cretica* can grow in coastal salt marshes of Karachi in Pakistan, and NaCl concentration in its seeds during germination can reach up to 850 mM. *Haloxylon stocksii* is a perennial with succulent stems and can tolerate 500 mM NaCl during germination*. Alhagi maurorum,* which thrives in salt marshes and coastal areas in Pakistan, is also considered one of the most important halophytic sources of biodiesel. *Arthrocnemum macrostachyum,* another perennial with succulent stems, has also been identified as a promising source of biodiesel [95]. These species, including *A. macrostachyum*, *C. cretica*, *H. stocksii*, and *A. maurorum*, contain approximately similar amounts of oil (25%, 23%, 23%, and 22%, respectively), but vary in their ash and sodium contents [95]. Another potential candidate for biofuel with positive prospects is the seashore mallow *Kosteletzkya pentacarpos*, an oilseed halophyte that grows on saline marginal lands [96], the seeds of which contain 18%–22% oil, which is comparable to cottonseed oil, which is edible, and shows some potential for biodiesel production [97]. *Kosteletzkya pentacarpos* can yield up to 1500 kg/ha of seeds, or up to 330 kg/ha of oil [86]. In addition, the Jerusalem artichoke (*Helianthus tuberosus*, or topinambour) is widespread in the coastal zones of China and is considered likewise one of the most promising sources of biomass energy. Jerusalem artichoke can yield 1–3.8 t/ha of algal diesel, which is much higher than the conventional oleaginous species such as castor, sunflower, soybean, and cotton, which yield 430–705 kg/ha of oil [98].

Fatty acid methyl esters from halophyte oils could be considered competitive with oils that are conventionally used for biodiesel production [95]. However, future research is necessary to investigate seed production of halophytes in detail, which is one of the most important criteria for the economic viability of their cultivation.

### Halophytes as resources for bioethanol

**Fig. 6:**
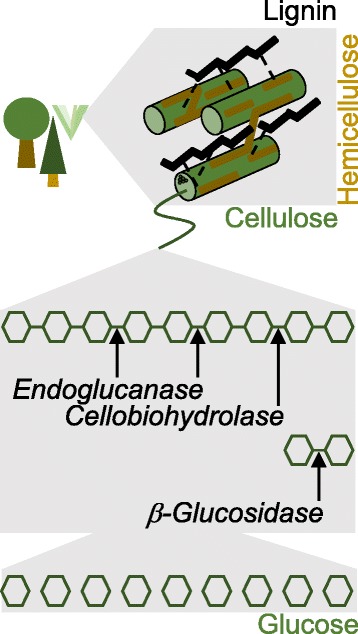
Components of plant cell wall including lignin, hemicellulose, and cellulose moieties. Cellulose degradation by a portfolio of cellulases is shown in a simplified form. Cellulose deconstruction yields monosacchardic glucose and small oligosaccharides by the synergistic action of three types of cellulases, namely endoglucanases, cellobiohydrolases, and β-glycosidases

Besides being a source of biodiesel, halophytes are also considered to be promising feedstock for producing bioethanol from the degradation of sugar-containing biomass. Some halophyte plants with biodiesel potential are for instance shown in Fig. 6. The most important prerequisite, beside rapid growth and high biomass productivity, is the production of releasable polysaccharides including intertwined cellulose and hemicellulose, which are embedded in the plant cell wall . Studies on the spatial distribution of halophytes have shown the potential of many such plants, e. g. about 365 species of perennial and annual halophytes in Iran were identified as a crucial source of bioethanol because they are rich in lignocellulose [17]. More than 30 years ago, the Nobel Laureate Melvin Calvin had recommended *Euphorbia tirucalli*, a succulent desert plant from East Africa, as a potential biofuel crop [99]. Other examples include halophytic forage plants, such as *Leptochloa fusca, Sporobolus virginicus*, and *Spartina patens*, which are rich in cellulose/hemicellulose and low in lignin, which can be fermented to ethanol after pre-treatment and enzymatic degradation. The highest amounts of hemicellulose (25%) and cellulose (29%) were recorded in *S. virginicus* [47].

*Halopyrum mucronatum* is a perennial grass with dimorphic seeds that thrives well under seawater irrigation. Biomass from this species contains approximately 37% cellulose, 28% hemicellulose, and 5% lignin [100]. *Desmostachya bipinnata* is a tall perennial grass found in inland and coastal areas of Sind province in Pakistan. Its seeds germinate at salinities as high as 400 mM NaCl [100]. Biomass from this species consists of nearly 26% cellulose, 24% hemicellulose, and 7% lignin. Plants with low lignin contents are preferred as feedstock of bioethanol because the cost of the additional steps required for the separation of lignin is minimized [95]. More interestingly, *Phragmites karka*, a perennial reed, is highly productive with seeds able to germinate under hypersaline conditions (500 mM NaCl) [95]. *Typha domingensis* is a rhizomatous perennial that can endure severe conditions such as moderate salinity and flooding; its biomass contains approximately 26% cellulose, 38% hemicelluloses, and 4% lignin, and *Panicum turgidum*, a perennial halophytic grass, can tolerate as high as 200 mM NaCl at the germination stage and produces biomass containing approximately 28% cellulose, 28% hemicellulose, and 6% lignin. In fact, *P. karka* and *P. turgidum*, irrigated with 100 mM and 125 mM of NaCl, respectively, maintain growth potentialities (in term of biomass production) comparable to plants grown on non-saline soils [74, 95]. These halophytes are rich in cellulose/hemicellulose and low in lignin, a composition suitable for efficient bioethanol production, and can thus strengthen the feedstock for biofuels [17, 74, 100, 101].

A recent study assessed *Juncus maritimus* a salt marsh plant that can be used for producing lignocellulosic biomass, because its total carbohydrate content can reach up to 73%, with cellulose and hemicellulose representing approximately 41% and 31%, respectively, of the lignocellulosic biomass [102]. *Tamarix aphylla*, irrigated with reclaimed sewage (EC approximately 3 dS/m^−1^) to different salinity levels or with brine (EC approximately 7–10 dS/m^−1^), produced 52 t/ha and 26 t/ha, respectively, of organic biomass. *Tamarix* was selected for its high cellulose content and low hemicellulose and phenol contents, properties particularly suitable for ethanol production, because the species of yeast commonly used for fermentation prefer C_6_^−^ sugars to C_5_^−^ sugars [99, 103]. In recent experiments, yield of *Tamarix* biomass, 18 months after transplanting, increased 60-fold, especially when it was irrigated with saline sewage (EC 8–10 dS/m^−1^ [97, 104].

However, polysaccharides must be released in all cases from their densely packed structure within the cell wall prior to enzymatic degradation, which implies efficient pre-treatment processes and highly effective and stable biocatalyst mixtures.

### Enzymatic degradation of plant cell wall

Sugars derived from plant feedstock can be used for producing chemicals and energy. Plant cell walls are composed of complex molecules, mainly lignocelluloses, which include cellulose, hemicellulose, and lignin [105]. The polysaccharides, namely cellulose and hemicellulose, can serve as sources of bioethanol but need to be separated from lignin prior to enzymatic decomposition. For efficient enzymatic degradation of plant-derived biomass, pre-treatment using a single agent or a combination of different techniques is indispensable to separate lignin from polysaccharidic residues. Yet, the outcomes of pre-treatment are often dependent on the chemical interactions between the pre-treatment agents and the plant cell-wall properties. In addition, some byproducts of pre-treatment may be inhibitory to cellulases and hemicellulases. These processes, which have been recently comprehensively reviewed [106], include a wide variety of pre-treatment techniques encompasses physical (grinding, fractionation, extrusion), chemical (acid, acid-acetone, alkaline, mid-alkali, and organosolv), physico-chemical (steam disintegration, hot water treatment, wet oxidation, fibre-mediated expansion), biological (microbial, fungal, and enzymatic) and other (microwave, thermo-expansion) methods. The available literature reports variable success in the halophytes submitted to pretreatment processes. A recent study was published on *Salicornia sinus-persica* for which the juice fraction was investigated for direct fermentation, and a hydrothermal pretreatment study was conducted on the fibre rich pulp fractions. Mild pre-treatment was performed to minimize energy inputs. The impact of the applied pre-treatment conditions was evaluated by studying the sugar recovery as well as the biomass fermentability. Wet fractionation yielded 70% juice and 30% pulp. Direct fermentation of the fresh juice using *S. cerevisiae* had no salt-induced inhibitory effect on the yeast and ethanol yields of about 70% were obtained. Cellulose convertibility of mildly pre-treated pulps was found to be significantly higher for severity factors over 2.00 with the best ethanol yield of 76.91 ± 3.03% was found at 3.06 [107]. In the halophyte plant *Juncus maritimus*, of the four fungal species (*Trichoderma* spp., *Aspergillus niger*, *Penicillium italicum* and *Fusarium* spp.) used as pre-treatment factors, *Trichoderma* spp. yielded the highest enzymatic activities of endoglucanase and beta-glucosidases [108]. In *Spratina argentiniensis*, different pre-treatments (phosphoric acid, ligninolytic enzymes and fungal supernatants) aimed to remove lignin and improving cellulose hydrolysis efficiency were assessed [109]. Results show that pretreatment with *Pycnoporus sanguineus* supernatant improved fermentable carbohydrates availability, yielding 56.84% cellulose hydrolysis. Finally, *Salicornia bigelovii* was reported to require only low hydrothermal pre-treatment [110].

### Enzyme portfolio to degrade plant cell wall

Based on biomass production worldwide, cellulose (30%–50%) is the most abundant polymer and hemicellulose (15%–35%) is the second most abundant polymer on earth, and both represent fully renewable sources. These polysaccharides are glued together and fixed by lignin, resulting in a densely packed structure stabilized through covalent and non-covalent linkages between cellulose, hemicellulose, and lignin [111]. Lignocellulose can be broken down by a number of glycoside hydrolases of prokaryotic or fungal origin [112]. Glycoside hydrolases specifically hydrolyse the glycosidic bonds between carbohydrates or between a carbohydrate and a non-carbohydrate moiety to produce monosaccharides that can be used for fermentation. Many enzymes have been isolated and characterized from various sources including gut bacteria from termites and ruminants, hot springs, compost heaps, and soil [4, 113–116].

Due to the complex structure of lignocellulose, a large portfolio of different glycoside hydrolases is needed to decompose the polysaccharides cellulose and hemicellulose. Three classes of cellulases act synergistically to degrade cellulose completely (Fig. 6) [111]. All enzymes thus catalyse the same reaction, namely the hydrolysis of beta-1,4 glycosidic linkages between glucose units in the linear cellulose chain. The endoglucanase attacks glycosidic bonds randomly at internal sites and produces short- and medium-sized oligosaccharides, including disaccharides and glucose. An exoglucanase (cellobiohydrolase) cleaves the β-1,4 bonds in such a way that the disaccharide cellobiose is released either from the reducing or the non-reducing end of the poly- and oligosaccharides. Finally, a β-glucosidase hydrolyses cellobiose to give two molecules of glucose. Different isozymes also separate glucose from short-length oligosaccharides such as cellotriose or cellotetraose [117, 118].

The assessment and valorisation of lignocellulosic biomass obtained from halophytic plants as feedstock for biofuel are receiving increasing attention from researchers [95]. Analysis of water extracts of halophyte biomass revealed high concentrations of salt ions including sodium and potassium, up to 150 mg/g and 18 mg/g (dry weight basis), respectively [19, 95]. The degree of salinity and the low concentrations of trace elements and phosphorous impair the growth of microbial communities that could be utilized for degrading halophytes. To decompose the salt-rich biomass derived from halophytes, an optimally adapted portfolio of enzymes with isozymes that are active under such conditions might serve as a promising approach to efficient production of sugars from these substrates.

### Examples of promising enzyme candidates

A variety of microorganisms colonize salty, hot, and dry soils that serve as ecological niches for halophytes [119]. Such microorganisms include prokaryotes and lower eukaryotes such as bacteria, Archaea, and filamentous fungi and can be assigned to the category of extremophilic microorganisms—microorganisms that thrive in environments that are considered extreme from the anthropogenic point of view. Beside truly halophilic microorganisms, there are many pro- and eukaryotic species that have been described as salt tolerant. These species are adapted to grow at high salt concentrations, but also survive without any salt in the medium, whereas true halophiles *require* salt for growth and prefer not only coastal dunes, hypersaline lakes, seas, salted foods, and saline deserts, but also halophytes that excrete and accumulate salts on the surface of their leaves [120].

Since extremophilic microorganisms thrive in the most inhabitable environments on the planet, it has been speculated that their enzymes are also capable of withstanding such conditions, which make the enzymes valuable under industrial conditions. Given their origin, enzymes from extremophilic microorganisms are called extremozymes [121]. Extremozymes from salt-tolerant microorganisms that can catalyse biochemical reactions over a wide range of salinity might be useful not only in the degradation of plant biomass derived from halophytes but also in many other harsh industrial processes involving highly concentrated salt solutions.

Only very few biomass-degrading enzymes have been isolated and characterized from true halophilic species. However, several cellulases and xylanases can tolerate extreme salinity and may have some potential as biomass degraders under such conditions (Table 3). Endoglucanase was obtained from the halophilic microorganism *Halomonas* sp. strain S66-4, a recombinant form, in a non-halophilic host *E. coli* and purified to homogeneity. The enzyme was salt tolerant up to 5 M of NaCl and yet retained more than 40% of its activity when tested directly in saline incubation mixtures [122].

**Table 3:** Some examples of salt-tolerant biomass-degrading enzymes and their properties

Enzyme	Species and strain	Salt tolerance	Optimum temperature (°C)	Reference
Halophilic species				
Cellulase	*Halomonas* sp. S66-4	5 M NaCl	45	[122]
Cellulase	*Haloarcula* sp. LLSG7	30% (*w*/*v*) NaCl	50	[157]
Cellulase	*Haloarcula* sp. G10	27.5% (*w*/*v*) NaCl	60	[158]
Xylanase	*Uncharacterized strain CL8*	5 M NaCl	60	[159]
Xylanase	*Uncharacterized strain CL8*	5 M NaCl	65	[159]
Xylanase	*Halorhabdus utahensis*	27%–30% (*w*/*v*) NaCl	55 and 70^a^	[160]
Salt-tolerant species				
Cellulase	*Aspergillus terreus* UniMAP AA-6	7.7% (*w*/*v*) NaCl	30	[161]
Cellulase	*Marinobacter* sp. MSI032	2% (*w*/*v*) NaCl	27–35	[162]
Xylanase	*Bacillus* sp. NTU-06	5% (*w*/*v*) NaCl	40	[163, 164]
Cellulase	*Stachybotrys microspora*	2.56 M NaCl	50	[123]
Cellulase	*Bacillus* sp. BG-CS10	2.5 M NaCl, 3 M KCl	55	[125]
Cellulase	Brine shrimp (*Artemia salina*)	600 mM NaCl	55	[164]
Cellulase	*Thalassobacillus* sp. LY18	10% (*w*/*v*) NaCl	60	[165]
Cellulase	*Bacillus agaradhaerens*	2 M NaCl, 0.8 M KCl	60 °C	[166]
Cellulase	*Thermoanaerobacter tengcongensis* MB4	3 M NaCl, 4 M KCl	75–80	[124]

^a^Two independent optima of activity were determined

A recent example of a salt-tolerant cellulase is the endoglucanase EG2 from the filamentous ascomycete *Stachybotrys microspora*. The enzyme displayed optimal activity at 850 mM NaCl and was active at concentrations up to 2.56 M and was considered highly suitable for producing bioethanol [123]. Several moderately salt tolerant and alkaliphilic ascomycetes were isolated from a Saharan salt flat (a *sabkha*) in southern Tunisia. Such *sabkha*s are colonized not only by lower eukaryotes or prokaryotes but also by halophytes. A single isolate of the genus *Penicillium* was found to secrete cellulases, indicating its potential to degrade plant cell walls of halophytes that inhabit identical environments. Such extracellular and extremely stable hydrolytic enzymes are common in extremophilic microorganisms, reflecting effective utilization of rare nutrient compounds in harsh environments.

Several salt-tolerant biomass-degrading enzymes are not derived from halophilic microorganisms at all (Table 3). An endoglucanase (Cel5A) from the thermophilic bacterium *Thermoanaerobacter tengcongensis* strain MB4 displayed almost 50% residual activity when incubated in the presence of 4 M KCl and 3 M NaCl. This microorganism thrives at a concentration of 0.03 mM of NaCl, at a lower ionic strength than that for optimal activity of the enzyme. Salt bridges are formed between amino acid carboxyl groups and sodium ions (R-COO^−^--Na^+^-−^−^OOC-R) to impart stability. Moreover, the enzyme Cel5A was stable in different ionic liquids used as solvents for degrading industrial cellulose [124]. Another bacterial endoglucanase was identified in the moderately salt-tolerant prokaryote *Thalassobacillus* sp. strain LY18. The enzyme was stable between 30 °C and 80 °C and in the presence of NaCl at concentrations up to 20% (*w*/*v*); moreover, based on its tolerance to organic solvents, the enzyme was the first endoglucanase to be described that is optimally active at high temperatures, high salinity, and at high pH values in organic solvents.

Only a few salt-active glycoside hydrolases have been described that have been genetically or biochemically modified to serve as efficient decomposers of biomass, e. g. a salt-dependent endoglucanase from the alkaliphilic microbial species *Bacillus* sp. strain BG-CS10. This bacterium can grow at a wide range of salt concentrations (0%–18% NaCl in the medium) and produces a large variety of extracellular glycoside hydrolases including xylanases, amylases, and mannanases. The cellulase CelB displayed a temperature profile that was regulated by salt concentration: the optimum temperature turned out to be 55 °C in the presence of 2.5 M NaCl but 35 °C when no NaCl was added, and enzymatic activity at 55 °C increased tenfold when 2.5 M NaCl or 3 M KCl was added to the reaction solution. The authors concluded that the thermostability of the enzyme can be controlled by adding NaCl [125].

Due to the complex structure of lignocellulosic materials, a portfolio of enzymes that are capable of acting in synergy is important for complete hydrolysis of polysaccharides and generation of fermentable sugars, which opens the way for the application of novel engineering techniques.

## Conclusion

Nowadays, the bulk of energy and products of the fuel and chemical industry are derived from fossil fuels. However, a transition from fossil-fuels-based industry to a widely applied bio-based industry is highly desirable in the energy and chemicals sectors. The potential of halophytes in biofuel production is reviewed here, including their characterization and the analysis of potential regions worldwide for their cultivation with focus on soil conditions. Specific species have been identified as sources of biodiesel and bioethanol, as well as enzymes to degrade the cell walls of those species. The review concludes by discussing several methods of producing bioethanol.

### Future research directions

#### Treated water and produced water for irrigating halophytes

Suitable ecosystems and halophyte habitats are highly dependent on different species and need be analysed in terms of those species. The comparative advantages and disadvantages of such species also need to be examined. Another approach is to investigate whether saline water in the form of treated wastewater or that produced during oil production can be used to irrigate halophytes. Further research is also required into these resources that are not linked at present to specific soils or habitats.

#### Side effects of cultivating halophytes

Halophytes for bioenergy need to be grown on a large scale if they are prove economically feasible and competitive with other energy sources. Such large-scale cultivation may have both positive and negative side effects, some of which are mentioned here.

A major question is the productivity of halophytes. Research is necessary to assess the productivity of halophytes accurately, since productivity is one of the most important criteria for the economic viability of halophyte cultivation.

In general, the costs of cultivating halophytes are comparable to those of conventional agriculture if we take into account the need to reduce CO_2_ emissions, to use arable land and freshwater resources carefully, and to reduce our dependence on oil. These considerations make halophytes more advantageous than algae, cyanobacteria, and organic waste, which are commonly used as feedstock for biofuel. Due to the fact that methanogenic microorganisms are outcompeted halophiles in salty environments, producing biofuel from biomass grown on salt-rich soils may contribute more to reducing the emissions of greenhouse gases than that grown on normal soils.

Mavi et al. [69] found that the loss of DOC from saline–sodic soils is lower than that from sodic soils because of bridging of cations at high electrolyte concentrations and suggest that increasing the concentration of electrolytes in sodic soils by liming or by irrigating with saline water may reduce the loss of nutrients through leaching and increase organic matter sequestration. However, as some regional environments may be particularly vulnerable and may be affected adversely by intensive agriculture, an analysis of specific local conditions and assessment of ecological impact are mandatory.

#### Improving bioenergy production from halophytes

Another future option would be to dissolve lignocellulose material from halophytes in saline ionic liquids, which are well established as alternative and ‘green’ solvents to be used in the pre-treatment of the walls of plant cells prior to enzymatic hydrolysis [126]. Lignocellulosic biomass from halophytes for ethanol production proved advantageous both in term of high net productivity and low maintenance costs [17]. Considering the advantages of second-generation biofuels, it is recommended that biofuel production be increased up to 10–20 EJ a year by 2050 [127] and the share of biofuels in the transport sector be increased from 3% to 8% worldwide between 2013 and 2035 [128].

## Abbreviations

CEC: Cation exchange capacity
DOC: Dissolved organic carbon
EC: Electrical conductivity
ECe: Electrical conductivity at reference water content
EMI: Electromagnetic induction
ET: Evapotranspiration
SOC: Soil organic carbon
TDS: Total dissolved salts
TE: Treated effluents
TOE: Tonnes of oil equivalent
